# Supplementary material for: Guided graph spectral embedding: Application to the C. elegans connectome
Source: Netw Neurosci. 2019 Jul 1;3(3):807–26. doi: 10.1162/netn_a_00084 (PMC6663470; doi:10.1162/netn_a_00084)
Supplement: Supplementary file 1 [file netn-03-807-s001.pdf]

Petrovic, M., Bolton, T.A.W., Preti, M.G., Liégeois, R., & Van De Ville, D. (2019). Supporting information for "Guided graph spectral embedding: application to the c. elegans connectome." *Network Neuroscience*, *3*(3), 807–826. https://doi.org/10.1162/netn_a_00084

SUPPORTIVE INFORMATION

Results of k-means clustering

In Supplementary Fig. S4, we present the proposed embedding from Figs. 4-6 and clusters of nodes with cooperation weight 1 derived by the k-means approach with 20 repetitions. Dimensionality of the considered data points was set to 2, *i.e.* entries of the two Slepian eigenvectors were used for clustering – the second and the third. The Silhouette method (Rousseeuw, 1987) was used to estimate the optimal number of clusters as the one which produces the minimal number of negative silhouette values. Convex hulls of each found cluster are represented by dashed black lines. The exact lists of neurons assigned to each cluster, for the three investigated cell types, are provided in Supplementary Tables 1, 2 and 3.

Table 1. Sensory neurons of the *C. elegans*. Columns correspond to clusters derived by optimized k-means.

C_1	C_2	C_3	C_4	C_5	C_6	C_7
AVM	CEPDR	AFDL	PHAL	ADEL	ADFR	ADFL
PDEL	CEPVR	AFDR	PHAR	ADER	ADLR	ADLL
PDER	IL1DR	ASEL	PHBL	ALMR	ASGL	ALA
PHCL	IL1R	ASER	PHBR	CEPDL	ASGR	ALML
PHCR	IL1VR	ASIL		CEPVL	ASHR	ALNL
PLML	IL2DR	ASIR		IL1DL	ASKL	ALNR
PLMR	IL2R	AWAL		IL1L	ASKR	AQR
PVM	IL2VR	AWAR		IL1VL	AWBL	ASHL
	OLLR	AWCL		IL2DL	AWBR	ASJL
	OLQVR	AWCR		IL2L		ASJR
	URXR			IL2VL		BAGL
				OLLL		BAGR
				OLQDL		FLPL
				OLQDR		FLPR
				OLQVL		PLNL
				URYDL		PLNR
				URYDR		PQR
				URYVL		PVDL
				URYVR		PVDR
						SDQL
						SDQR
						URXL

Evaluation of The Clustering

In order to evaluate the inspected clusters of sensory neurons (Fig. 4B), interneurons (Fig. 5B) and motoneurons (Fig. 6B), we used statistical testing of communities (clusters). In all three cases, the nodes with importance $m_i = 0$ are considered as one additional cluster. We use the Newman-Girvan modularity as statistic (M. E. J. Newman, 2006). A vector of nodal assignments to clusters expresses its goodness of fit to the underlying adjacency matrix through the value of modularity Q. It is calculated as:

Table 2. Interneurons of the *C. elegans*. Columns correspond to clusters derived by optimized k-means.

C_1	C_2	C_3	C_4	C_5	C_6
AVBL	AIML	AIBL	AIAL	ADAL	AVAL
AVBR	AIMR	AIBR	AIAR	ADAR	AVAR
AVG	AVFL	AINL		AVEL	AVDL
AVJL	AVFR	AINR		AVER	AVDR
AVJR	AVHL	AIYL		AVKL	LUAL
BDUL	AVHR	AIYR		AVKR	LUAR
BDUR	PVQL	AIZL		DVA	PVCL
PVPR	PVQR	AIZR		DVC	PVCR
	RIFL	AUAL		PVPL	PVR
	RIFR	AUAR		PVT	PVWL
		RIAL		RICL	PVWR
		RIAR		RICR	
		RIBL		RIGL	
		RIBR		RIGR	
		RIH		RIPL	
		RIR		RIPR	
				RIS	
				RMGL	
				RMGR	
				URBL	
				URBR	

$$Q = \frac{1}{2w} \sum_{i,j}^{N} ([\mathbf{A}_{bin}]_{i,j} - \frac{d_i d_j}{2w}) \delta_{C_i, C_j},$$

where N is the number of nodes, w is the total strength of edges in the graph, $\mathbf{A}_{bin}$ is the graph binary adjacency matrix, d_i denotes the degree of the ith node, δ is the Kronecker delta function, and C_i denotes the cluster to which the ith node belongs.

In Supplementary Fig. S8, we present the results of the statistical approach for the case of sensory (red plots), inter- (grey plots) and motoneurons (green plots). The modularity values for the assignments to clusters as found by k-means clustering are marked with the dashed lines and labeled with Q_{sensory}, $Q_{\text{inter-}}$, and $Q_{\text{moto-}}$ (Supplementary Fig. S8B). For the number of clusters estimated by the Silhouette method, we generated 999 random assignment vectors and calculated Q each time, in order to build a null distribution (Supplementary Fig. S8A).

As Q_{sensory}, $Q_{\text{inter-}}$, and $Q_{\text{moto-}}$ are above the corresponding distributions of modularity for random assignments, we conclude that the found clustering is significant. Since these modularity values are strictly greater than all other Q values for random assignments, and, consequently, from any chosen percentile of the calculated distributions, the test rejects the null hypothesis that the chosen clustering is random at even very small significance levels. Finally, we note that the distribution of Q in the case of interneurons is slightly closer to the corresponding value of $Q_{\text{inter-}}$ than in the case of sensory or

Table 3. Motoneurons of the *C. elegans*. Columns correspond to clusters derived by optimized k-means.

C_1	C_2	C_3	C_4	C_5	C_6	C_7
AS10	DD04	DD05	DA04	AVL	AS11	AS01
AS06	VA07	VA08	DB03	DA08	DA09	AS02
AS07	VB06	VA09	DB04	DB07	DD06	AS03
AS08	VB07	VB08	DD02	PVNR	DVB	AS04
AS09	VD07	VB09	DD03	RID	PDA	AS05
DA07	VD08	VD10	VA06	RIML	PDB	DA01
DB05		VD09	VB02	RIMR	VA11	DA02
DB06			VB03	RIVL	VA12	DA03
HSNL			VB04	RIVR	VB10	DA05
PVNL			VB05	RMDDL	VB11	DA06
RMHL			VC01	RMDDR	VD12	DB01
RMHR			VC02	RMDL	VD13	DB02
SABD			VC03	RMDR		DD01
SABVL			VD02	RMDVL		HSNR
SABVR			VD03	RMDVR		VA01
SIADL			VD04	RMED		VA02
SIADR			VD05	RMEL		VA03
SIAVL			VD06	RMER		VA04
SIAVR				RMEV		VA05
SIBDL				RMFL		VB01
SIBDR				RMFR		VC04
SIBVL				SAADL		VD01
SIBVR				SAADR		
SMBDR				SAAVL		
URADL				SAAVR		
VA10				SMBDL		
VC05				SMBVL		
				SMBVR		
				SMDDL		
				SMDDR		
				SMDVL		
				SMDVR		
				URADR		
				URAVL		
				URAVR		
				VD11		

motoneurons. This can be expected, since interneurons are more strongly connected to other cell types, and thus, do not impose as strong communities as for the clusters formed from sensory or motoneurons.

Supplementary Figures

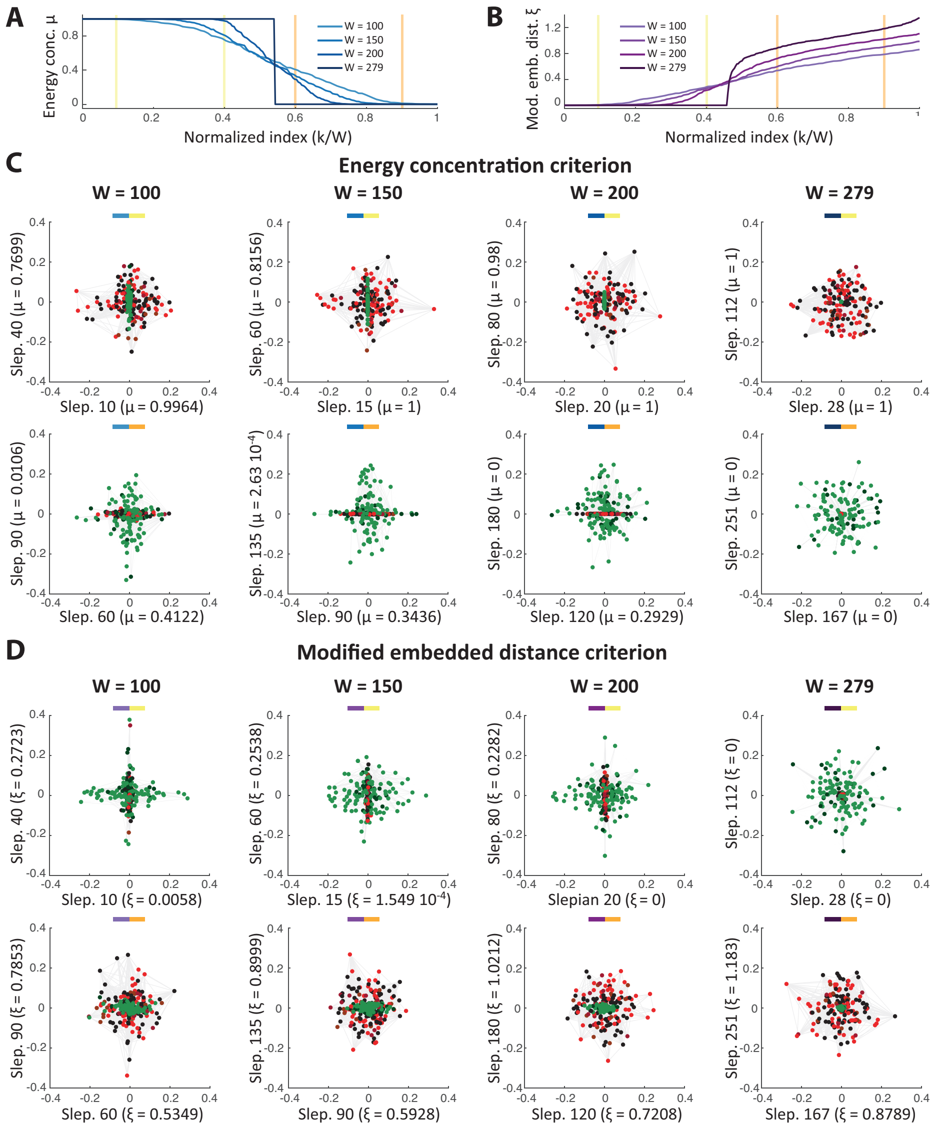

Figure S1. For energy concentration (**A**) and modified embedded distance (**B**) criteria, eigenspectra at bandwidth $W = 100, 150, 200, 279$, as depicted by increasingly darker blue or purple shades, respectively. Yellow and orange vertical bars map locations of the eigenspectra at which Slepian vectors are shown (**C** and **D**). They are displayed for increasing bandwidth going from left ($W = 100$) to right ($W = 279$, full bandwidth). For energy concentration (**C**), the first row illustrates two Slepian vectors mapping the start of the spectrum (normalized indices of 0.1 — strongly concentrated in $\mathcal{S}$ — and 0.4 — still concentrated, but less for lower bandwidth). The second row denotes two Slepian vectors from the second half of the spectrum (normalized indices of 0.6 — mildly concentrated in $\mathcal{S}$ using a smaller bandwidth — and 0.9 — not concentrated at all). Visualizations are similar for modified embedded distance (**D**), but in this case, low eigenvalues imply either non-concentrated (*e.g.*, X axis, first row of plots) or mildly concentrated but low localized spatial frequency Slepian vectors (for instance, Y axis, first row of plots, $W = 200$), while high eigenvalues relate to high localized spatial frequency Slepian vectors (see Y axis, second row of plots). See Van De Ville et al. (2017a) for another preliminary analysis of the dataset from the modified embedded distance viewpoint. μ and ξ values of the shown Slepian vectors are provided in parentheses on each axis.

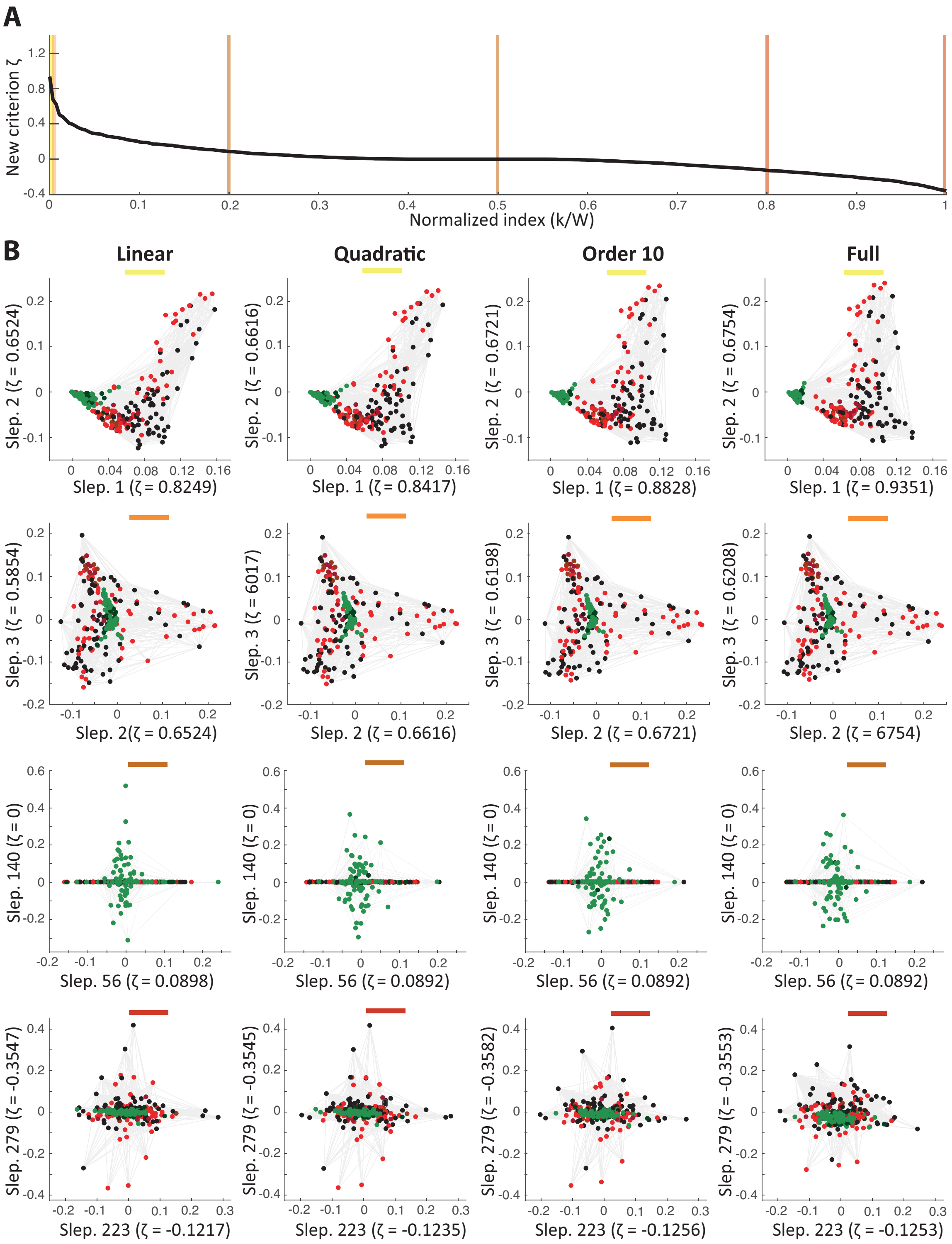

Figure S2. Eigenspectrum of the newly developped ζ criterion (**A**), with vertical bars highlighting the locations of the spectrum at which four pairs of Slepian vectors were sampled for display (**B**, from first to fourth row as respectively depicted by yellow, orange, brown and red color codes). Results obtained with linear, quadratic and order 10 approximations, as well as from a full computation of $\mathbf{M} - \mathbf{L}^{1/2}\mathbf{M}\mathbf{L}^{1/2}$, are respectively shown from left to right. ζ values of the shown Slepian vectors are provided in parentheses on each axis.

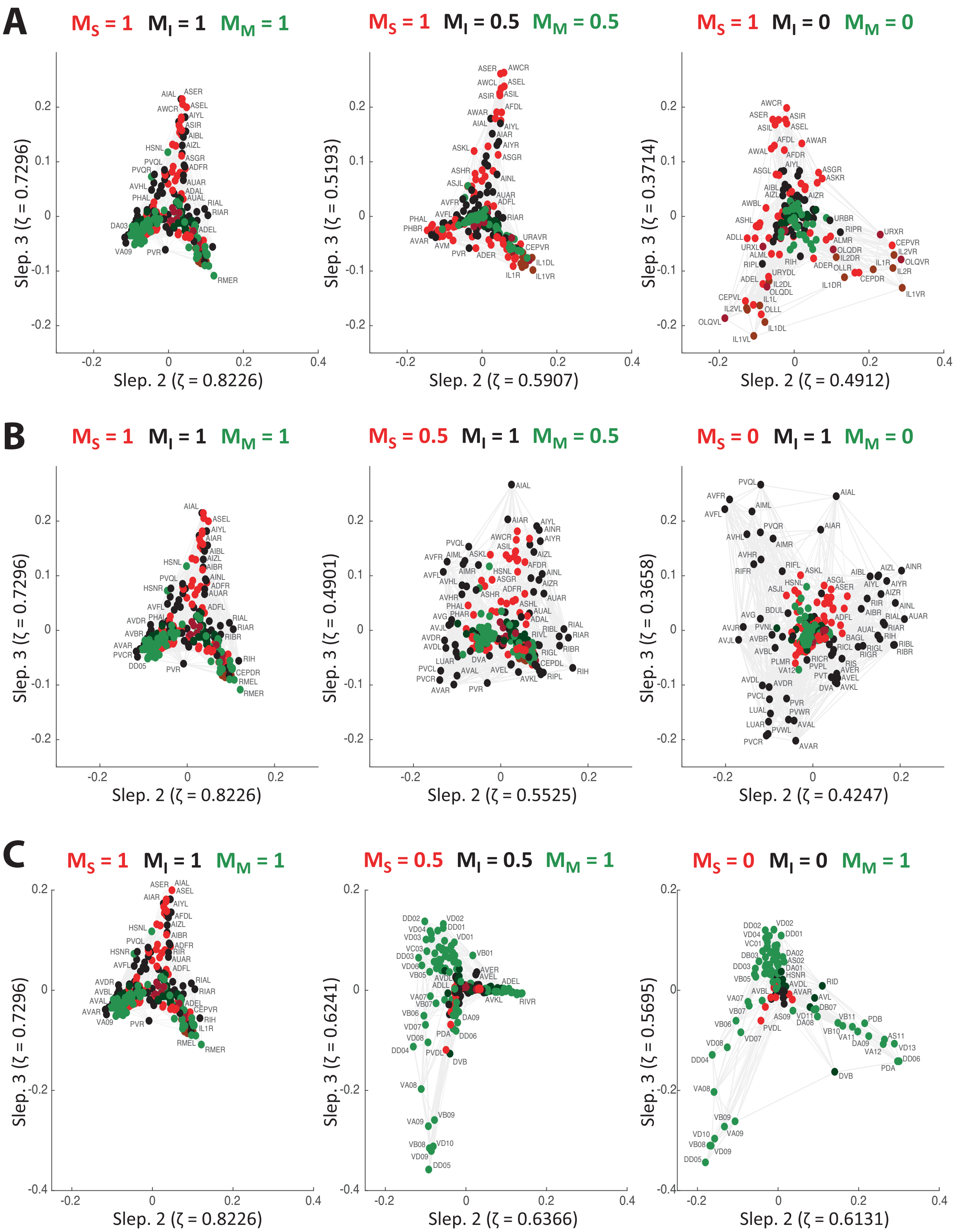

Figure S3. Start (left column), intermediate (middle column) and end (right column) representations of sensory neuron (**A**), interneuron (**B**) or motoneuron (**C**) trajectories, respectively, setting cooperation weights for other neuron types to 1, 0.5 or 0. Cells are labeled according to Varshney et al. (2011). The start representation is the same across cases, since then $\mathbf{M} = \mathbf{I}$ and the problem boils down to the eigendecomposition of the adjacency matrix $\mathbf{A}$, or equivalently of the Laplacian $\mathbf{L} = \mathbf{I} - \mathbf{A}$ highlighted in Fig. 1.

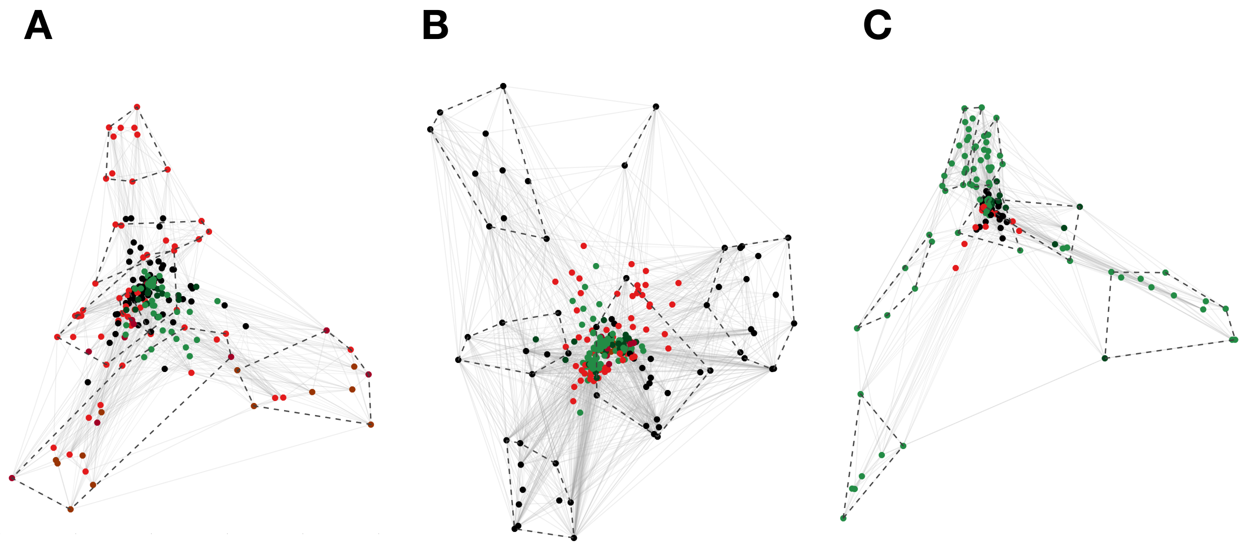

Figure S4. Clusters derived by repeated k-means clustering of the focused nodes in the case of sensory neurons (**A**), interneurons (**B**) or motoneurons (**C**). The optimal number of clusters was estimated with the Silhouette approach. Nodes constituting the border of each cluster's convex hull are connected by a dashed black line to visualize the clusters.

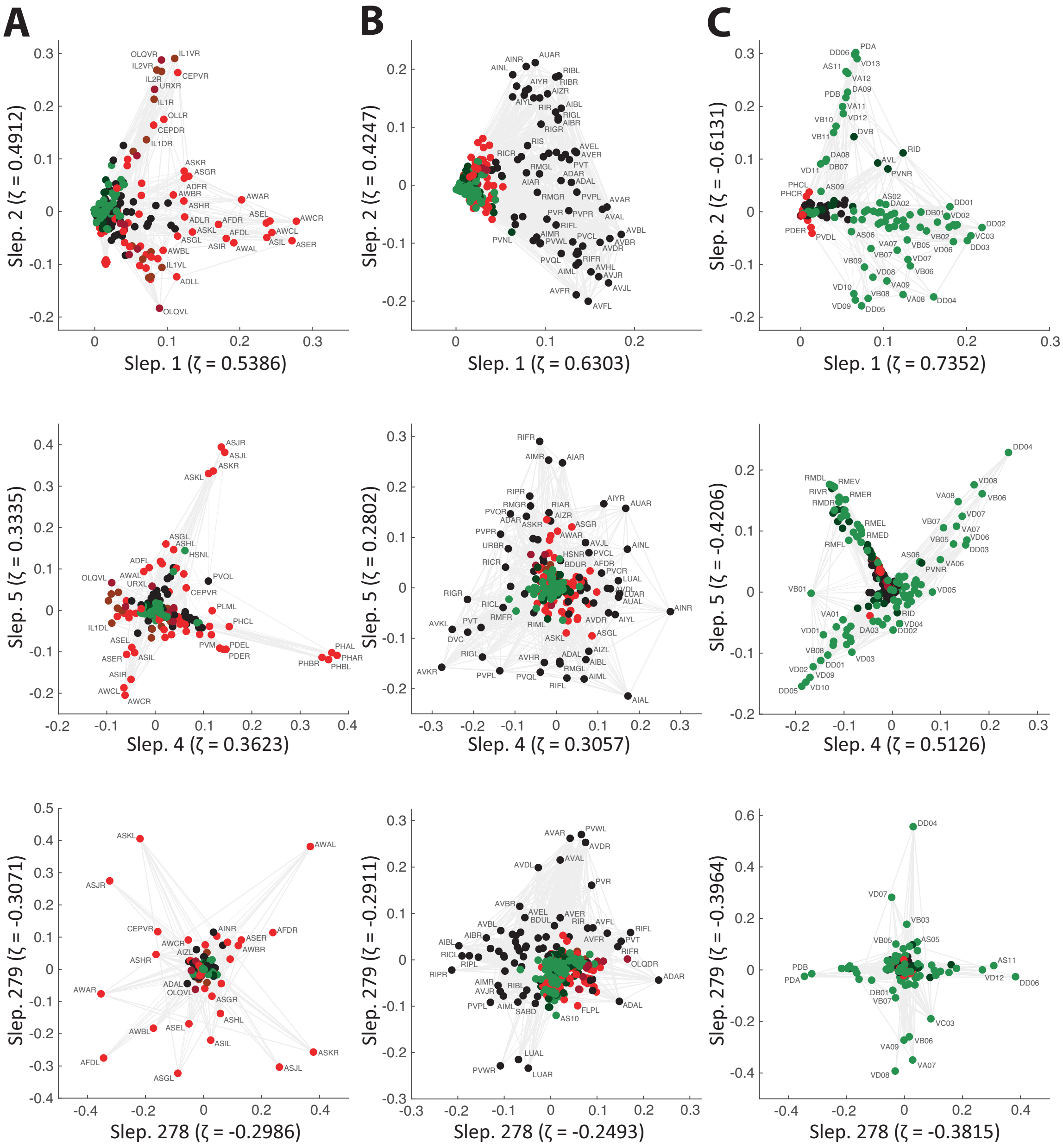

Figure S5. Focussing on (**A**) sensory neurons (red), (**B**) interneurons (black) or (**C**) motoneurons (green), two-dimensional visualization using alternative sets of Slepian vectors: first and second (first row), fourth and fifth (second row), or last two (third row). Cells are labeled according to Varshney et al. (2011). ζ values of the shown Slepian vectors are provided in parentheses on each axis.

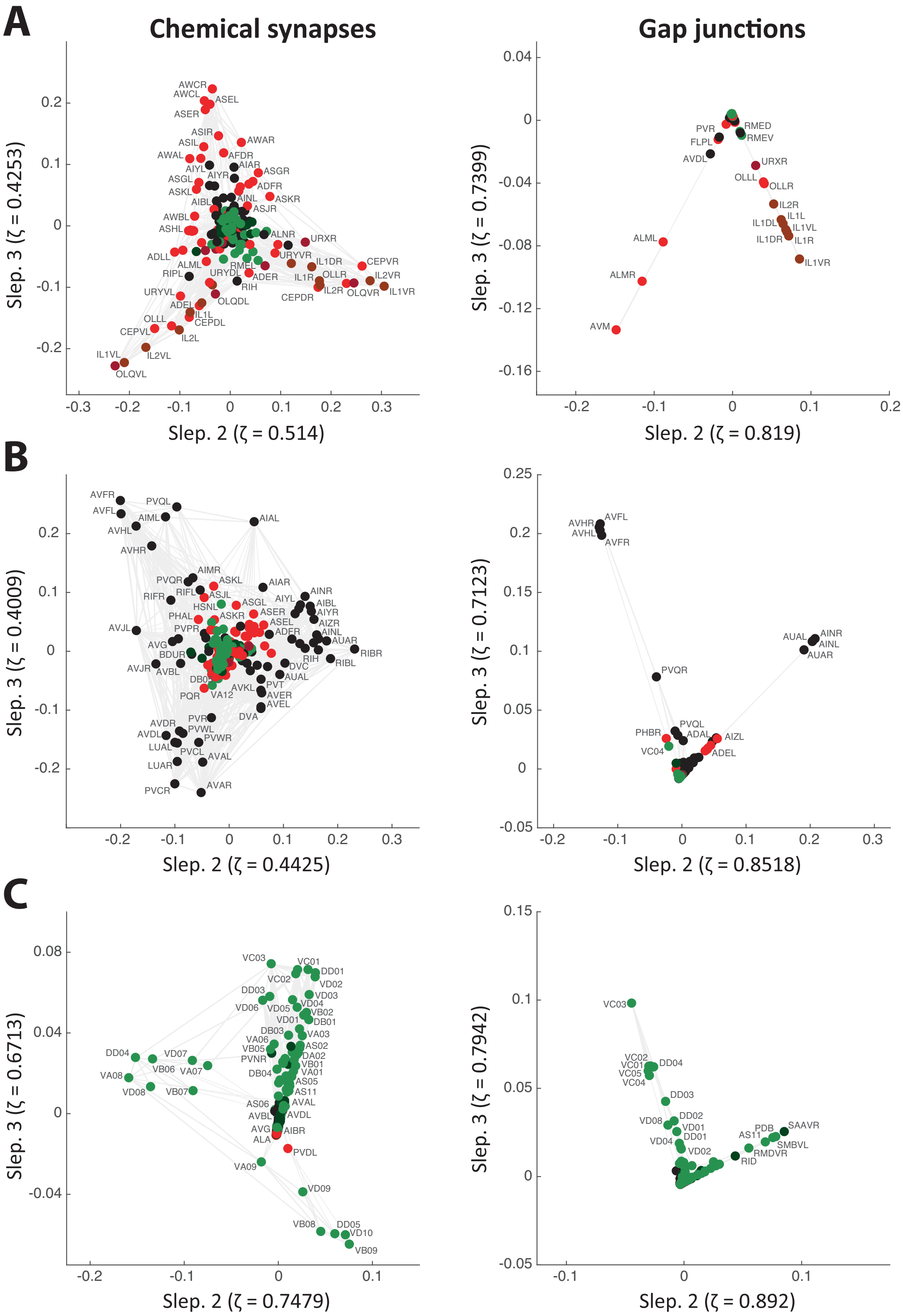

Figure S6. Separate two-dimensional visualizations when only considering chemical synapses (left column) or gap junctions (right column) for sensory neurons (**A**), interneurons (**B**) or motoneurons (**C**). Cells are labeled according to Varshney et al. (2011). ζ values of the shown Slepian vectors are provided in parentheses on each axis.

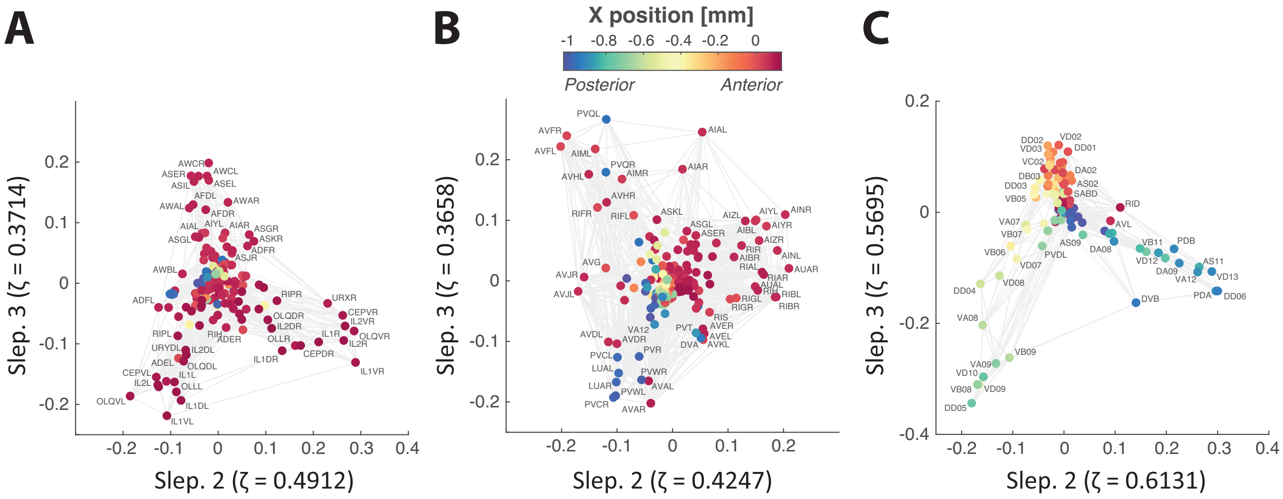

Figure S7. Two-dimensional visualizations for sensory neurons (**A**), interneurons (**B**) or motoneurons (**C**) when representing each neuron as a function of its position along the X direction. A value of 0 indicates the location of the nerve ring, and positional data was retrieved from Varier and Kaiser (2011). ζ values of the shown Slepian vectors are provided in parentheses on each axis.

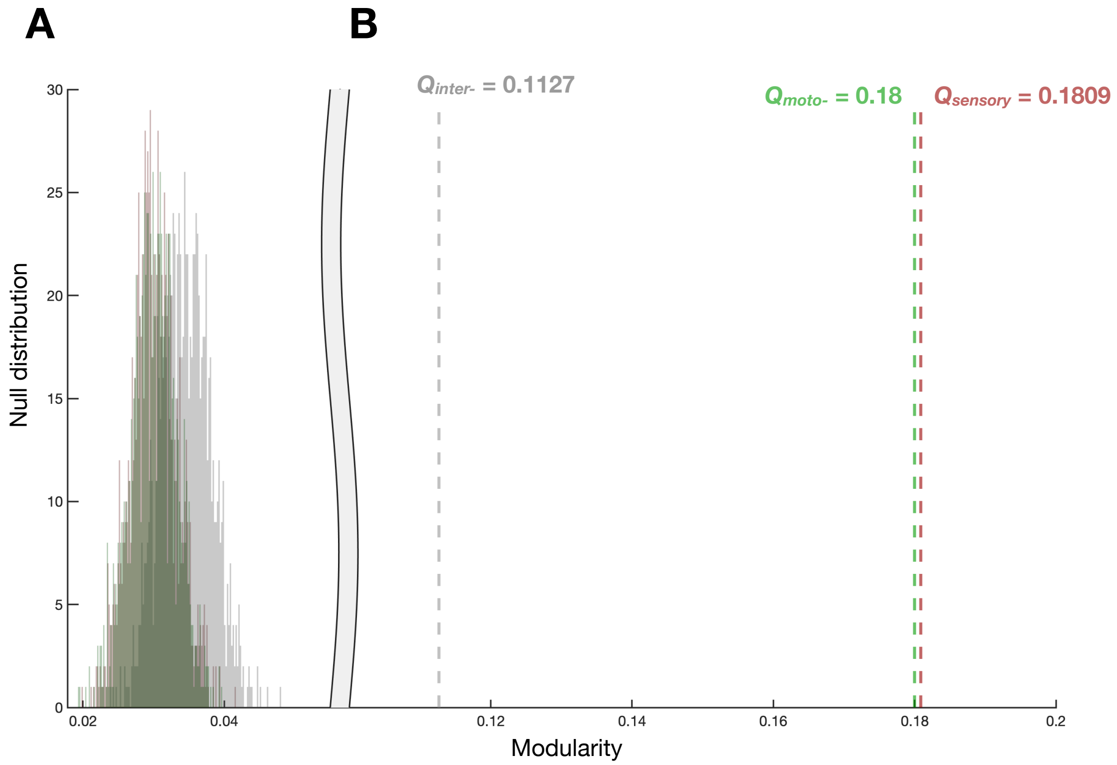

Figure S8. Testing of clustering assignments when the focus is on sensory neurons (red), interneurons (grey) or motoneurons (green). For each case, all nodes of other types are considered as one additional cluster. The null distributions of the modularity of random assignments to clusters are given on the left side of the plot (**A**).The dashed straight lines on the right represent values of modularity Q for the clusters in Fig. S4 derived by the k-means approach (**B**). The x-axis is broken at 0.06 for better visualization.
